# The Impact of Diabetes on Exercise Tolerance in Patients After Cardiovascular Events

**DOI:** 10.3390/jcm14155561

**Published:** 2025-08-07

**Authors:** Beata Czechowska, Jacek Chrzczanowicz, Rafał Gawor, Aleksandra Zarzycka, Tomasz Kostka, Joanna Kostka

**Affiliations:** 1 Department of Methodology of Teaching Movement, Medical University of Lodz, Pl. Hallera 1, 90-647 Lodz, Poland; beata.czechowska@umed.lodz.pl (B.C.); aleksandra.zarzycka@stud.umed.lodz.pl (A.Z.); 2 Cardiac Rehabilitation Centre, Copernicus Memorial Hospital, Popioly 40, 93-438 Lodz, Poland; jacekchrz@interia.pl (J.C.); gawor@onet.eu (R.G.); 3 Department of Geriatrics, Healthy Ageing Research Centre (HARC), Medical University of Lodz, Pomorska 251, 92-213 Lodz, Poland; tomasz.kostka@umed.lodz.pl; 4 Department of Physioprophylaxis, Medical University of Lodz, Pl. Hallera 1, 90-647 Lodz, Poland

**Keywords:** diabetes, exercise tolerance, cardiac rehabilitation

## Abstract

**Background**: Diabetes mellitus (DM) is a significant factor affecting prognosis and functional capacity in patients after cardiovascular events. This study aimed to assess the impact of coexisting diabetes on exercise tolerance and hemodynamic parameters in patients qualified for cardiac rehabilitation. **Methods**: A total of 452 patients (86 women, 366 men; mean age 63.21 ± 7.16 years) who had experienced cardiovascular incidents, including 226 individuals with coexisting DM (DM group) and 226 age- (±1 year) and sex-matched individuals without DM (non-DM group), were included in the analysis. All participants underwent an exercise test using a bicycle ergometer. Clinical data, comorbidities, medication use, left ventricular ejection fraction, and exercise test parameters were evaluated. **Results**: Patients with DM displayed a higher number of comorbidities (4.29 ± 1.26 vs. 3.19 ± 1.30; *p* < 0.001), greater medication use (8.71 ± 2.16 vs. 7.83 ± 2.05; *p* < 0.001), higher body mass (86.93 ± 13.35 kg vs. 80.92 ± 15.25 kg; *p* < 0.001), and a lower left ventricular ejection fraction (48.78 ± 8.99% vs. 50.01 ± 8.40%; *p* = 0.002) compared to those in the non-DM group. Diabetic patients also exhibited lower exercise capacity, expressed as peak power per kilogram of body mass (1.05 ± 0.27 W/kg vs. 1.16 ± 0.31 W/kg; *p* < 0.001). No significant differences were observed regarding absolute peak power or maximum heart rate. **Conclusions**: In patients after cardiovascular incidents, the presence of diabetes is associated with reduced relative exercise capacity and lower ejection fraction.

## 1. Introduction

Diabetes mellitus (DM) is one of the leading causes of mortality worldwide, with its prevalence continuing to rise steadily [1,2,3]. Data indicate that chronic disturbances in blood glucose levels affect millions of people globally. In 2019, nearly 463 million individuals worldwide were diagnosed with DM, and this number continues to grow. According to projections by the International Diabetes Federation, the global prevalence is expected to reach approximately 700 million individuals by 2045 [4,5]. According to data from combined national sources, including the national health insurance system, the NATPOL epidemiological study, the RECEPTOmetr Sequence study, and regional childhood diabetes registries, it is estimated that the number of people with DM in Poland is 2.68 million, of whom 0.51 million are unaware of their condition [6].

Diabetes induces numerous pathological changes that significantly impair patients’ quality of life. Individuals with DM exhibit higher mortality rates due to cardiovascular diseases, including atherosclerosis-related complications such as myocardial infarction and heart failure [1,3,7].

Patients with DM have twice the risk of experiencing cardiovascular events, primarily due to factors such as hypertension, dyslipidemia, and other diabetes-related consequences that promote the development of atherosclerosis [5,8]. According to the Polish Lipidogram 2015 study, the prevalence of DM in individuals without cardiovascular disease was 10.6%, whereas in those with cardiovascular disease it was 31% [9]. Diabetes accelerates the progression of atherosclerosis and increases the risk of myocardial infarction, heart failure, and cardiovascular death [10,11]. Reduced exercise capacity is one of the most important predictors of mortality [12]. The physiological response to physical exertion in patients with coexisting DM may be impaired due to vascular damage, autonomic neuropathy, impaired venous return, and hyperglycemia-related metabolic disturbances. Furthermore, patients with DM often experience so-called “silent ischemia”, which can hinder the accurate interpretation of exercise test results and increase the risk of undetected complications [13].

There is a paucity of data assessing the influence of DM on exercise tolerance in cardiac patients. Therefore, the aim of this study was to determine whether the co-occurrence of DM affects exercise tolerance and exercise test outcomes in comparison to the results for age- and sex-matched non-diabetic individuals with a history of cardiovascular events.

## 2. Materials and Methods

The results of exercise tests and medical records of 452 patients (86 women, 366 men) from the Cardiology Department of the Early Cardiac Rehabilitation Center in Łódź were analyzed. All patients were qualified for phase II cardiac rehabilitation in an inpatient setting. Inclusion was based on the following criteria: age over 18 years, qualification for stationary rehabilitation, availability of complete medical documentation, and results of exercise stress tests. Patients under 18 years of age, as well as those with missing or invalid stress test results, were excluded. The study included patients admitted to the facility between 2019 and 2022.

The study posed no risk to participants, and written consent was obtained from the head of the medical facility. The research was approved by the Bioethics Committee of the Medical University of Łódź under reference number RNN/17/24/KE, dated 9 January 2024.

A total of 226 individuals (43 women, 183 men) with cardiovascular incidents and coexisting DM (confirmed by medical records) were included in the study group (DM group). The control group of 226 individuals matched for age (±1 year) and sex (43 women, 183 men) after cardiovascular events but without a documented diagnosis of DM (non-DM group) were consecutively recruited.

To assess physical performance and exercise tolerance, all participants underwent a graded exercise test on an ErgoSelect 100P cycle ergometer with continuous ECG monitoring using a Cardiovit AT-2 Plus system. The exercise test was performed for clinical purposes and training planning as part of cardiac rehabilitation.

The exercise test followed a ramp protocol, with incremental workload increases of 25 W every 3 min, starting from an initial load of 50 W [14,15]. The test consisted of several phases, beginning with a 2–3 min warm-up without resistance, followed by incremental loading starting at 50 W.

Each subsequent stage involved a gradual increase in workload by 25 W. Exercise intensity was measured in watts (W). The final recovery phase (cool-down) was performed in a seated position.

The degree of exercise-induced fatigue, as subjectively assessed by the patient, was measured using the 15-point Borg scale, where the patient indicated a score from 6 (no fatigue) to 20 (maximum fatigue) [16,17].

Based on the exercise test reports, reasons for test termination were distinguished, including patient exhaustion, heart rate limit, blood pressure response (systolic blood pressure exceeding 250 mmHg and/or diastolic blood pressure exceeding 115 mmHg or a drop in systolic pressure below 90–100 mmHg) [16,18], cardiac arrhythmias, abnormalities on the ECG record, chest pain (angina), dyspnea, and lower limb pain. The test was considered terminated when the subject reached 85% of the predicted maximum heart rate, calculated using the following formula [16,18]:

HRmax = (220 − age in years) × 85%

The heart rate limit was further lowered by 10% in patients taking beta blockers.

Based on the exercise test results, the following parameters were analyzed [19,20,21,22,23]:

- Peak power output—the maximum workload achieved during the test [W].
- Peak power output relative to body mass—peak workload expressed in watts per kilogram of body mass [W/kg].
- Resting heart rate (HR rest)—heart rate before the start of the exercise test.
- Peak heart rate (HR peak)—maximum heart rate achieved during the exercise test.
- Heart rate recovery (HRR)—the rate at which the heart rate normalizes after exercise, expressed as the difference between peak heart rate at exercise termination and heart rate measured after 1 min of recovery [19,23]:
  HRR = HRpeak − HR1min
- Systolic (SBP) and diastolic (DBP) blood pressure measurements—resting and peak values recorded during the test.
- Reasons for exercise test termination.
- Resting double product (DP rest)—product of resting heart rate and resting systolic blood pressure [20,21,22].
- Exercise double product (DP exercise)—product of peak heart rate and peak systolic blood pressure during the test [20,21,22]. Since the product of systolic blood pressure and heart rate yields a large numerical value, this index was divided by 100 for analytical purposes [21,22].
  DP=(SBP×HR)/100
- Double product reserve (DP reserve)—an indicator of the heart’s ability to increase oxygen demand during physical exertion compared to resting conditions [21].
  DP reserve=DP exerciseDP rest

Additionally, medical records were reviewed to collect sociodemographic and anthropometric data (body mass and height, based on which BMI was calculated), as well as information on comorbidities, number of medications taken, left ventricular ejection fraction, and blood morphology parameters (hemoglobin level, red blood cell count, hematocrit, etc.). Some variables displayed missing data, which were limited in scope and generally not related to the primary outcome measures. Specifically, time since the cardiac event was unavailable for 3 participants in the DM group and 6 in the non-DM group; ejection fraction (EF) was missing for 4 and 2 participants, respectively; selected blood markers (hemoglobin, red blood cell count, hematocrit) were missing for 10 and 6; HRR for 1 participant in the DM group; and the number of medications for 2 participants in the non-DM group. More substantial missing data were observed only for ratings on the Borg scale (18 and 10 missing values in the DM and non-DM groups, respectively).

### Statistical Analyses

The data obtained during the study were recorded using Microsoft Excel 2019, and statistical analysis were performed using Statistica version 13.3 (Statsoft, Kraków, Poland). The results are presented as mean ± standard deviation, along with minimum and maximum values. To assess the normality of distribution, the Shapiro–Wilk test was performed. Comparisons between groups for quantitative variables were conducted using the Student’s *t*-test or the Mann–Whitney U test, and for categorical variables using the Chi-square test. For statistically significant results for quantitative variables, effect sizes were calculated using the standardized test statistic (Z) according to the formula $r=Z/\sqrt{N}$, where Z is the value of the test statistic, and N is the total number of observations in both groups. Effect sizes were interpreted as small (0.1), medium (0.3), and large (0.5). The minimal clinically important difference (MCID) for relative exercise capacity (W/kg) was estimated using a distribution-based approach, defined as 0.5 times the standard deviation of baseline values, in accordance with established methods described by Sedaghat [24]. Statistical significance was set at *p* < 0.05.

## 3. Results

The study group consisted of 452 patients who had experienced cardiovascular incidents, including 86 women and 366 men (Table 1). Based on the primary diagnosis at hospitalization, patients were categorized into the following groups: ST-elevation myocardial infarction (STEMI, 38.7%), non-ST-elevation myocardial infarction (NSTEMI, 33.4%), coronary artery bypass grafting (CABG, 19.5%), valve replacement (4.4%), and other causes (4%).

The treatment approaches within the study group included percutaneous coronary interventions (72%), surgical procedures (25%), and other treatment methods (3%).

Statistically significant differences between groups were observed in body mass and BMI values (*p* < 0.001). Patients with DM displayed higher values than those without DM. Specifically, in the DM group, the mean body mass was 86.93 ± 13.35 kg and the BMI was 29.12 ± 3.73 kg/m^2^, compared to 80.92 ± 15.25 kg and BMI of 27.19 ± 3.98 kg/m^2^ in the non-DM group (Table 1), with effect sizes of 0.23 and 0.25 for body weight and BMI, respectively.

The study group was also assessed for comorbidities, excluding cardiovascular diseases, as DM was the primary inclusion criterion for one of the groups. The comorbidities included degenerative joint diseases, rheumatoid arthritis, chronic gastrointestinal diseases, chronic kidney disease, stroke, peripheral arterial disease, thyroid diseases, eye diseases, hearing loss, gout, osteoporosis, epilepsy, asthma, and sleep apnea. The most frequently diagnosed conditions were degenerative joint disease (23.0%), chronic kidney disease (14.4%), and chronic gastrointestinal diseases (11.3%). However, no significant differences in the prevalence of these conditions were observed between patients with and without DM.

The average number of comorbidities was significantly higher in the DM group compared to the non-DM group (4.29 ± 1.26 vs. 3.19 ± 1.30; *p* < 0.001), with an effect size of 0.39. Diabetic patients also used a greater number of medications (8.71 ± 2.16 vs. 7.83 ± 2.05; *p* < 0.001), with an effect size of 0.22, and had a lower left ventricular ejection fraction (48.78 ± 8.99% vs. 50.01 ± 8.40%; *p* = 0.002), with a small effect size (0.14). No statistically significant difference was observed in regards to peripheral blood counts or smoking status, both current and past. A near-significant difference in time since the cardiovascular event (39.41 vs. 34.94 days, *p* = 0.05) was observed between DM and non-DM patients (Table 1). However, the effect size was very small (0.09).

The analysis of exercise test parameters, including exercise tolerance, is presented in Table 2. Peak power output in absolute terms did not differ between the DM and non-DM groups. However, when adjusted for body mass, the power output achieved by the DM group was significantly lower compared to that for the non-DM (*p* < 0.001), with an effect size of 0.18, indicating a small effect. The MCID was determined to be 0.15 W/kg, using a distribution-based approach (0.5 × SD, with SD = 0.30). The observed difference between groups was 0.11 W/kg, suggesting that the effect, while statistically significant, may be not clinically meaningful. A trend toward a higher resting heart rate was observed in the DM group (*p* = 0.08). No significant differences were found between the groups regarding the remaining exercise test parameters.

The reasons for terminating the exercise test among diabetic and non-diabetic patients are presented in Table 3. The most common reasons for test termination were general fatigue (n = 250), reaching the target heart rate (n = 144), and lower limb pain (n = 105). No statistically significant differences were found between groups in relation to the reasons for test termination. However, a tendency was observed for more frequent test termination due to lower limb pain in diabetic patients (60 vs. 45; *p* = 0.095).

## 4. Discussion

In the present study, the impact of DM on exercise tolerance indicators was assessed in cardiac patients. Patients were matched for age and sex to exclude major confounding factors that could potentially distort the interpretation of results. In absolute terms, exercise tolerance, as reflected by peak workload achieved during the exercise test, did not differ significantly between groups. However, when normalized to body mass (W/kg), peak workload was significantly lower in the group with concomitant DM (1.05 vs. 1.16 W/kg; *p* < 0.001). Relative values (W/kg) are considered a more sensitive indicator of functional exercise capacity than are absolute values, particularly in populations with differences in body mass and composition. Previous studies have shown that the peak workload-to-weight ratio (PWR) represents a significant prognostic indicator, surpassing the predictive value of absolute workload. For example, Yasui et al. demonstrated that patients with chronic heart failure and lower PWR exhibited a higher risk of adverse outcomes, even after adjustment for confounding variables [25]. In our study, despite a statistically significant between-group difference in relative power output (Δ = 0.11 W/kg), the observed effect did not exceed the threshold for clinical significance. MCID was predefined at 0.15 W/kg. As the between-group difference fell below this threshold, the clinical relevance of the intervention effect is questionable, suggesting that the magnitude of difference may not be perceptible or meaningful in a real-world clinical context. Thus, the change is statistically significant, but not clinically significant. The limitation of exercise capacity in patients with DM may depend not so much on the diagnosis itself, but on potentially occurring complications of diabetes, glycemic control, the severity of the disease, or the coexistence of diseases to which the development of diabetes contributes [26]. Future studies should be designed in a way that allows for the analysis of their impact on exercise tolerance. The course of DM itself may limit exercise capacity through integrated pathophysiological mechanisms, i.e., myocardiogenic, myogenic, vasculogenic and neurogenic types [26].

Even if the difference is not clinically significant, it is worth noting that DM predisposes patients to limited exercise capacity, and these patients in particular should be included in intervention programs. Lower relative exercise capacity results in higher physiological strain during daily activities (greater body mass burden), which may be associated with reduced quality of life and increased risk of recurrent cardiovascular events. Under exercise test conditions performed on a cycle ergometer (as in the present study), where the influence of body mass on the workload is limited, the actual physiological burden related to daily activities may be underestimated. Future studies would benefit from comparing the results of exercise tests performed on a treadmill in similar patient groups.

Patients with DM were characterized by significantly higher body mass (86.93 vs. 80.92 kg; *p* < 0.001) and higher BMI values (29.12 vs. 27.19 kg/m^2^; *p* < 0.001). The mean BMI in the DM group is borderline between overweight and obesity. Studies indicate a J-shaped relationship between BMI and mortality risk, with a BMI above 30 kg/m^2^ clearly associated with a poorer prognosis [27]. Increased body mass in individuals with DM may also be associated with a higher proportion of fat mass at the expense of lean muscle mass (sarcopenic obesity). Although body composition was not specifically analyzed in this study, previous research has highlighted the bidirectional relationship between DM and sarcopenia—sarcopenia may increase the risk of developing DM, and vice versa [28]. According to literature data, the prevalence of sarcopenia in patients with DM may be up to three times higher than in patients without DM and is associated with a poor prognosis [29]. Such alterations in body composition can significantly affect exercise tolerance. According to existing reports, overweight and obesity negatively affect physical performance in patients after cardiovascular events, reflected by poorer results in assessments such as the six-minute walk test or the Short Physical Performance Battery (SPPB) [30,31]. Iris den Uijl et al. demonstrated that BMI significantly affects physical activity levels, with obese patients performing fewer steps per minute compared to those with normal body mass (6.39 vs. 7.50 steps/min; *p* = 0.01) [32]. Beyond muscle-related factors, numerous other mechanisms may contribute to the negative impact of DM on exercise capacity, including metabolic disturbances, microvascular dysfunction, neurological impairments, and myocardial abnormalities [26]. For these reasons, people with DM are encouraged to be physically active and to minimize sedentary behavior. According to recommendations from scientific organizations (including the American Diabetes Association, the American College of Sports Medicine, and Diabetes Canada), individuals with DM should engage in both aerobic and resistance training. These forms of exercise, especially when combined, not only help improve DM management but have also been associated with lower rates of cardiovascular and all-cause mortality among people with diabetes who are more physically active compared to those who are inactive. However, when planning activity for people with DM, the risk of hypoglycemia should be considered, and the initial load/activity time should be lower than the recommended levels [33].

A significantly lower left ventricular ejection fraction (EF) was also observed in the DM group (47.88% vs. 51.01%; *p* = 0.002). Reduced EF may reflect impaired cardiac function in diabetic patients, potentially resulting from microvascular complications of DM or a higher prevalence of diabetic cardiomyopathy. Similarly, a study by Nishitani et al. demonstrated lower EF values in patients with DM compared to non-DM individuals (59.70% vs. 65.30%) [34]. The DM group also exhibited a significantly greater number of comorbidities (*p* < 0.001), which may further contribute to reduced exercise capacity and increased risk of complications during cardiac rehabilitation. However, it is important to note that none of the individual comorbidities analyzed were significantly more prevalent in the DM group compared to in the non-DM group.

A relatively high proportion of participants in both groups reported current smoking: 27.34% in the DM group and 33.60% in the non- DM group. Although this difference did not reach statistical significance, the overall prevalence of smoking remains concerning, given its well-established role as a major cardiovascular risk factor. Importantly, the coexistence of DM and smoking has been shown to exert a synergistic effect on cardiovascular risk. As demonstrated by Yang et al., individuals with both DM and a history of smoking exhibited a significantly higher risk of cardiovascular events compared to that of non-smokers without DM, with the combination of both factors being associated with a 2.45-fold increased risk (HR = 2.45; 95% CI: 2.24–2.68). This finding underscores the importance of targeted smoking cessation efforts, particularly among individuals with DM undergoing cardiac rehabilitation [35].

The reasons for termination of the exercise test were also analyzed. No significant differences were observed between groups in this regard. However, there was a trend towards statistical significance concerning lower limb pain as a reason for test termination (*p* = 0.095), which may be associated with potential vascular, neurological, or muscular abnormalities commonly observed in diabetic patients [26]. Nevertheless, reliable conclusions in this area require further investigation.

This study has several limitations. We did not present data regarding the severity of DM, its duration, treatment modality, glycemic control, HbA1c levels, or the presence of diabetic complications, which would have allowed for a broader understanding of its relationship with exercise capacity. These data were not available in the medical records of patients treated at the cardiac rehabilitation center. It should be noted that DM severity may correlate with exercise limitations more strongly than DM presence alone. Furthermore, the DM group exhibited a higher number of comorbidities and medications taken, which may have influenced the results. Another limitation is the lack of analysis of the medications taken, both diabetic and cardioactive. Patients with DM also exhibited a slightly longer time interval since the cardiovascular event, although this difference approached but did not reach statistical significance (*p* = 0.05), and the effect size was small (0.09). Finally, the exercise stress test was conducted using a bicycle ergometer. This method may reflect patients’ functional abilities less accurately than the treadmill test. However, at the rehabilitation center, tests are performed to determine the appropriate load for aerobic (interval) training on a stationary bicycle, which justifies the use of this method.

Future studies should consider the severity of DM, its duration, treatment modality, and the presence of complications. Due to the risk of coexisting sarcopenic obesity in patients with DM, it would be valuable to include not only basic anthropometric indicators (body weight, BMI) but also body composition, particularly the proportion of muscle tissue, in future analyses. It is also worthwhile to compare the results of ergometer tests with those of treadmill or walking tests, which will allow for a better assessment of actual functional capacity. Future studies should also include a more detailed assessment of patients’ functional status to better characterize limitations in diabetic cardiac patients.

## 5. Conclusions

The results of the study confirm that the presence of diabetes in patients after cardiovascular events is associated with a negative impact on exercise tolerance. The group of patients with diabetes was characterized by lower peak power output during exercise testing, normalized to body mass, although it should be emphasized that this difference did not reach a clinically significant level. Additionally, reduced left ventricular ejection fraction and higher body mass and consequently, higher BMI values, were observed. These factors may significantly limit exercise tolerance and affect the effectiveness of cardiac rehabilitation.

The findings of this study possess practical implications for cardiac rehabilitation planning. Patients with DM require a more tailored approach that addresses not only improvements in cardiovascular performance but also focuses on body mass reduction and enhancements in muscular strength and endurance. Exercise programs should be individualized and designed to improve relative performance indicators, not merely absolute workload. Relying solely on absolute values from exercise testing may underestimate the true extent of functional limitations in patients with diabetes.

## Figures and Tables

**Table 1:** Characteristics of the study group, N = 452, $\bar{x}$ ± SD (min–max).

Variable	**All Patients** $\bar{x}$ ± SD (Min–Max) or n (%)	**Non-DM Group** $\bar{x}$ ± SD (Min–Max) or n (%)	**DM Group** $\bar{x}$ ± SD (Min–Max) or n (%)	*p*-Value
Sex [women/men]	452 (86/366)	226 (43/183)	226 (43/183)	1.000
Age [years]	63.21 ± 7.16 (44–86)	63.29 ± 7.09 (45–85)	63.13 ± 7.25 (44–86)	0.82
Height [m]	1.72 ± 0.08 (1.52–1.94)	1.72 ± 0.08 (1.52–1.94)	1.73 ± 0.08 (1.52–1.92)	0.49
Body mass [kg]	83.93 ± 14.63 (50–140)	80.92 ± 15.25 (50–140)	86.93 ± 13.35 (58–140)	<0.001
BMI [kg/m^2^]	28.16 ± 3.97 (18.96–45.19)	27.19 ± 3.98 (18.96–42.59)	29.12 ± 3.73 (20.66–45.19)	<0.001
Number of comorbidities [n]	3.74 ± 1.39 (0–8)	3.19 ± 1.30 (0–7)	4.29 ± 1.26 (1–8)	<0.001
Number of medications taken [n]	8.27 ± 2.15 (3–17)	7.83 ± 2.05 (3–17)	8.71 ± 2.16 (3–16)	<0.001
EF [%]	49.89 ± 8.76 (20–71)	51.01 ± 8.40 (20–70)	48.78 ± 8.99 (20–71)	0.002
Time since cardiovascular event [days]	37.17 ± 24.22 (3–188)	34.94 ± 22.42 (3–124)	39.41 ± 25.75 (5–188)	0.05
Hemoglobin [g/dl]	13.29 ± 1.45 (9.30–17.50)	13.35 ± 1.47 (9.30–17.50)	13.22 ± 1.42 (9.30–16.90)	0.27
Red blood cells (×10^6^/μL)	4.41 ± 0.46 (3.26–5.88)	4.41 ± 0.45 (3.30–5.62)	4.41 ± 0.46 (3.26–5.88)	0.98
Hematocrit [%]	39.41 ± 3.95 (28.20–51.00)	39.51 ± 4.00 (29.3–51.00)	39.30 ± 3.91 (28.20–51.00)	0.52
Smoking in the past [n/%]	156 (34.5%)	72 (28.6%)	84 (32.81%)	0.24
Current smoking [n/%]	156 (34.5%)	86 (33.60%)	70 (27.34%)	0.11

DM—diabetes mellitus; EF—ejection fraction; BMI—body mass index.

**Table 2:** Characteristics of selected exercise test parameters; $\bar{x}$ ± SD (min–max).

Variable	**All Patients** $\bar{x}$ ± SD (Min–Max)	**Non-DM Group** $\bar{x}$ ± SD (Min–Max)	**DM Group** $\bar{x}$ ± SD (Min–Max)	*p*-Value
Borg scale [pts]	14.25 ± 2.04 (7–19)	14.32 ± 2.05 (7–19)	14.19 ± 2.04 (7–17)	0.32
Peak power [W]	91.66 ± 25.83 (30–179)	92.67 ± 26.66 (33–179)	90.65 ± 24.99 (30–164)	0.41
Peak power per kg of body mass [W/kg]	1.11 ± 0.30 (0.32–2.10)	1.16 ± 0.31 (0.41–2.10)	1.05 ± 0.27 (0.32–2.02)	<0.001
HR rest [bpm]	72.25 ± 11.12 (44–113)	71.31 ± 11.51 (44–113)	73.18 ± 10.66 (48–108)	0.08
HR peak [bpm]	110.48 ± 15.46 (70–191)	109.39 ± 14.67 (70–154)	111.58 ± 16.18 (71–191)	0.13
HRR [bpm]	29.28 ± 12.88 (−31–96)	28.57 ± 12.22 (−31–77)	29.99 ± 13.49 (−12–96)	0.24
SBP rest [mmHg]	115.39 ± 15.07 (82–159)	115.33 ± 14.52 (89–158)	115.45 ± 15.63 (82–159)	0.97
DBP rest [mmHg]	70.03 ± 9.21 (47–106)	69.42 ± 8.85 (47–91)	70.66 ± 9.53 (47–106)	0.28
SBP max [mmHg]	161.92 ± 24.95 (85–230)	162.55 ± 24.83 (85–230)	161.29 ± 25.11 (102–230)	0.59
DBP max [mmHg]	79.32 ± 11.37 (49–147)	79.55 ± 11.46 (51–147)	79.09 ± 11.30 (49–115)	0.67
DP rest/100	83.41 ± 17.37(42.50–151.42)	82.37 ± 17.95 (49.84–151.42)	84.44 ± 16.73 (42.50–144.72)	0.21
DP exercise/100	179.76 ± 40.00 (69.70–364.08)	179.04 ± 40.54 (69.70–269.10)	180.48 ± 39.52 (79.52–364.08)	0.70
DP reserve	2.21 ± 0.55 (0.79–4.24)	2.22 ± 0.54 (0.79–4.09)	2.19 ± 0.56 (1.01–4.24)	0.53

DM—diabetes mellitus; HR—heart rate; HRR—heart rate recovery; SBP—systolic blood pressure; DBP—diastolic blood pressure; DP—double product.

**Table 3:** Reasons for terminating the exercise test in the study group.

Reasons for Terminating the Exercise Test	All Patients	Non-DM Group	DM Group	*p*-Value
Fatigue [n/%]	250/55.3	121/53.5	129/57.1	0.449
HR limit [n/%]	144/31.9	74/32.7	70/31.0	0.687
Blood pressure limit [n/%]	19/4.2	12/5.3	7/3.1	0.241
Rhythm disturbances [n/%]	17/3.8	6/2.7	11/4.9	0.216
ECG abnormalities [n/%]	19/4.2	12/5.3	7/3.1	0.241
Lower limb pain [n/%]	105/23.2	45/19.9	60/26.5	0.095
Other [n/%]	22/4.9	11/4.9	11/4.9	1.00

DM—diabetes mellitus; HR limit—heart rate limit; ECG—electrocardiogram.

## Data Availability

The statistical data used to support the presented findings may be obtained by sending a request to the corresponding author, due to privacy constraints.

## Abbreviations

The following abbreviations are used in this manuscript:

DBP	Diastolic Blood Pressure
DM	Diabetes Mellitus
DP	Diabetes Mellitus
EF	Ejection Fraction
HRpeak	Peak Heart Rate
HRrest	Resting Heart Rate
HRR	Heart Rate Recovery
MCID	Minimal Clinically Important Difference
PWR	Workload-to-Weight Ratio
SBP	Systolic Blood Pressure
SPPB	Short Physical Performance Battery
